# Development of New Recombinant DgK Antigen for Diagnosis of *Dirofilaria immitis* Infections in Dogs Using ELISA Technique and Its Comparison to Molecular Methods

**DOI:** 10.22034/ibj.22.4.283

**Published:** 2018-07

**Authors:** Rahmat Solgi, Seyed Mahmoud Sadjjadi, Mehdi Mohebali, Zabihollah Zarei, Majid Golkar, Abbasali Raz

**Affiliations:** 1 Department of Parasitology and Mycology, School of Medicine, Shiraz University of Medical Sciences, Shiraz, Iran; 2 Department of Medical Parasitology and Mycology, School of Public Health, Tehran University of Medical Sciences, Iran; 3 Center for Research of Endemic Parasites of Iran (CREPI), Tehran University of Medical Sciences, Tehran, Iran; 4 Molecular Parasitology Laboratory, Department of Parasitology, Pasteur Institute of Iran, Tehran, Iran; 5 Malaria and Vector Research Group (MVRG), Biotechnology Research Center (BRC), Pasteur Institute of Iran, Tehran, Iran

**Keywords:** *Keywords*: Proteins, Enzyme-linked immunosorbent assays, *Dirofilaria immitis*, Dirofilariasis

## Abstract

**Background:**

*Dirofilaria immitis* is a cosmopolitan zoonotic, vector-borne parasite of carnivorous animals causing dirofilariasis in human beings. Common commercial serodiagnostic tests for canine dirofilariasis usually lead to different results in their sensitivity and specificity. The present study reports development of recombinant *DgK* (rDgK) antigen of *D. immitis* for accurate immunodiagnosis of *D. Immitis*-infected dogs using indirect ELISA test.

**Methods:**

The r*DgK* coding sequence was successfully sequenced, codon optimized and cloned into pET-24a(+) expression vector and then expressed in *Escherichia coli*. The recombinant *DgK* was affinity purified using Ni²^+^-charged HiTrap chelating column, followed by testing in Western blotting and enzyme-linked immunosorbent assays (ELISA) with dog sera from a dirofilariasis endemic area. The performance of rDgK ELISA was evaluated using 60 sera collected from suspected dogs, while molecular technique was used as a reference test.

**Results:**

Sera from positive control *D. immitis* infection produced a strong IgG antibody response to rDgK both in ELISA and Western blotting tests. The sensitivity and specificity related to diagnostic potential of rDgK for ELISA were 92.5% and 87.5%, respectively. The results of rDgK ELISA showed a high agreement (0.764) with molecular identification.

**Conclusions:**

The findings revealed that the developed new rDgK antigen is sensitive and specific for immunodiagnosis of canine dirofilariasis using ELISA test.

## INTRODUCTION

Dirofilariasis, a cosmopolitan zoonotic infection with increasing reports over the last decades, is caused by *Dirofilaria immitis*[1]. Due to global warming, the prevalence of dirofilariasis among canine and human is increasing in many parts of the world, including Iran[2-4]. The importance of canine dirofilariasis is heightened by the challenges involved in its diagnosis as well as its pathogenic and zoonotic potentials[5].

*D. immitis* inadvertently causes pulmonary dirofilariasis in human, which is characterised by the presence of pulmonary nodules containing larvae[6]. Moreover, other types of human dirofilariasis caused by *D. Immitis*, including ophthalmic disease, have also been reported[7]. Current control methods for human dirofilarisis are focused on diagnosis and treatment of canine dirofilariasis. The ELISA and immune-chromatographic test system have been proved to be clinically useful for diagnosis of canine heartworm[8]. The development of a novel serological diagnostic test for better diagnosis of canine and human dirofilariasis is critical to disrupt life cycles and to avoid invasive diagnostic procedures, especially in human[9]. A number of diverse native and recombinant antigens have been used in serological diagnostic tests[10-12]; however, it has been revealed that the recombinant antigens are more sensitive and specific than crude antigens[13]. The major surface antigen of *D. immitis* infective larvae, previously characterized in its native form[14], has already been produced as a recombinant antigen[15]. Di35, as an immunodominant antigen, is encoded by *Dg2* gene.

It has been shown that *D. immitis* worms have another similar gene named *DgK*. The exonic regions of these two genes are highly conserved with 91% sequence identity[16]. Until now, no published evidence exists concerning the antigenic characteristics of *DgK*. The present study was designed to develop recombinant *DgK* (r*DgK*) and its potential value for serodiagnosis of *D. immitis* infection in dogs using an ELISA technique.

## MATERIALS AND METHODS

### Parasite and sera samples preparation

**Fig. 1:**
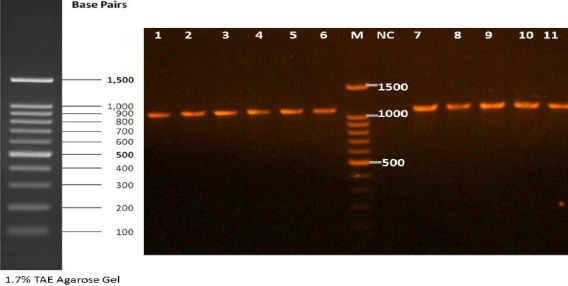
Gel electrophoresis of *Dirofilaria immitis* PCR products. Lanes 1-11, positive isolates amplified 1100-bp PCR products; lane M, DNA ladder; lane NC, negative control.

Fresh adult female and male worms of *D. immitis* were obtained from a 14-year-old dog (Ardabil, Iran) based on the previous techniques[17]. To collect the microfilariae (mff), the distal uterine portions were removed from the live gravid females and incubated in 0.9% saline solution at 37°C for 1-2 h. Live mff were collected from the incubated solution by centrifugation at 6000 ×g for 2 min. The adult worms and collected mff were stored in RNAlater stabilizing solution (Qiagen, Hilden, Germany) and kept at -80°C until RNA extraction. The dog serum samples used in the present study included 30 positive and 30 negative sera. The positive sera, confirmed by morphological and PCR methods, were obtained from dogs naturally infected with *D. immitis* in an endemic region; the sera were kindly provided by Tehran University of Medical Sciences, Tehran, Iran. However, the negative sera were collected from healthy pet dogs of a non-endemic area referred to a small animal hospital of University of Tehran with no mff in their blood samples and no history and clinical signs of canine dirofilariasis. For clinical serodiagnostic potential of rDgK ELISA, 60 serum samples from dog suspected for *D. immitis* infection with the signs of occasional cough, unusual intolerance to exercise, fainting spells, and cachexia were collected from Meshkin Shahr city of Ardabil Province, an endemic region of *D. immitis* in the north west of Iran[18]. All the samples were examined by morphological methods as well as by PCR (Fig. 1) and ELISA. For determination of sensitivity of rDgK ELISA in view of the time points after infection, 15 young dogs suspected to dirofilariasos (<2 years old) were included.

The mff of each sample was counted as described previously[19]. The Knott’s concentration technique (KCT) was applied to concentrate and detect the mff of *D. immitis*[20]. Briefly, 1 ml anticoagulated blood was mixed with 10 ml of 2% formaldehyde, followed by centrifugation at 168 ×g for 5 minutes. The supernatant was discarded, and a drop of methylene blue stain was added to the sediment. The sediment was transferred onto the center of slide for examination under a 10× microscope. Morphometric analysis of mff such as body length and diameter and the form of front end and the tail was conducted to distinguish and differentiate between *D. immitis*, *D. repens*, and *Acnthocheilonema* *spp*. based on previous studies[21,22]. The amplification of internal transcribed spacer region 2 (ITS2) was performed using specific primer DiF (5′-CATTATC GAGCTTCAACAAACAAC-3′) and DiR (TTCAGC GGGTAATCACGACTG). The amplification was performed as described previously[23].

### Plasmids, bacterial strains, and growth condition

*E. coli* strains DH5α and BL21 (DE3) and the pTG19-T (Invitrogen, Barcelona, Spain) and pET-24a(+) (Biomatik, Canada) plasmids were used for cloning and recombinant protein expression, respectively. Bacterial cells were grown in LB agar and LB broth (Merck, Darmstadt, Germany) and incubated at 30°C overnight and 4 h, respectively. Final concentrations of 50 µg/ml and 100 µg/ml of kanamycin and ampicillin were used for cloning procedure and protein expression, respectively.

### Cloning procedures

Total RNA was extracted from adult male and female worms and from mff of *D. immitis* using RNA Mini Kit (Qiagen, USA) according to the manufacturer’s instruction. Reverse transcription was performed using RevertAid™ First Strand cDNA Synthesis Kit (Thermo Fisher Scientific, USA) and processed based on the instruction provided by the manufacturer. Two specific primer DgKF (5′-ATGAGAAGCGTACGGGATACAGG-3′) anf DgKR (CCGTATTGATTTTGCGACGCG) were designed using the only available sequence of *DgK* cDNA sequence from *D. immitis* in NCBI database (accession number: AB004253). The amplified fragment was cloned into the pTG19-T vector using the TA Cloning Kit (SinaClon, Tehran, Iran) following the manufacturer’s instruction and transformed into DH5α competent cells. The recombinant plasmid pTG19-T-*DgK* was confirmed by DNA sequencing.

### Codon optimization and gene synthesis

The nucleotide sequence of the *DgK* ORF (open reading frame), determined in this study, was deposited in the GenBank (accession number: KU058149). This sequence was used for codon optimization and gene synthesis. The *DgK* sequence was codon optimized, chemically synthesized and directly inserted into the expression vector pET-24a(+) between Bam*HI* and Eco*RI* restriction sites (Biomatik, Canada).

### Protein expression and purification

BL21 (DE3) competent cells were transformed with the recombinant expression vector pET-24a(+)-*DgK*. For the expression, transformed competent cells were grown in LB broth medium (5 ml) supplemented with kanamycin (50 mg/L). When the optical density (OD) 600_nm_ reached at 0.6, the protein expression was induced by addition of 1 IPTG (1 mM fnal concentration), at 30°C for 4 h. The expression of rDgK protein was analyzed by SDS-PAGE using a 12% gel. The His tagged rDgK protein was purified using 5 ml Ni²^+^-charged HiTrap chelating column (Bio-Rad, Hercules, CA, USA) according the manufacture’s instruction and then dialyzed against phosphate buffered saline at pH 7.4 and stored at -20ºC until use. Concentration of the purified protein was determined by Bradford Protein Assay Kit (Bio-Rad) using bovine serum albumin (BSA) as standard.

### Western blot analysis

For determination of immune-reactivity, the purified rDgK protein was initially separated by SDS-PAGE (12%) and then transferred onto a nitrocellulose membrane (0.2 m, Bio-Rad). The membrane was blocked in BSA solution (2%) t at 4°C overnight. *D. immitis*-infected and uninfected dog sera (1:100 diluted) were used as priary antibodies in the blocking buffer (Tris-buffered saline containing 0.1% [v/v] Tween-20 [TBST] and BSA [2%]). A goat anti-dog IgG-HRP conjugate was used as the secondary antibody. The blot was visualized using diaminobenzidine (TIANGEN, Beijing, China), as the substrate [24].

### ELISA

Checkboard titration was performed to optimize the concentration of recombinant antigen (1 µg/well) and serum samples (100 µl of 1:100 dilution), goat anti-dog IgG-HRP-conjugated secondary antibody (100 µl of 1:5000 dilution), and substrate solution (100 µl of O-Phenylenediamine Dihydrochloride, Amresco, USA). Absorbance was recorded using a microplate reader (BioTek, USA) at 450 nm. The cut-off values were achieved as the mean ± three standard deviations (SD) of negative control sera (30 healthy pet dogs) from non-endemic areas.

### Sensitivity and specificity of the rDgK ELISA

Sixty sera from dogs suspected of *D. immitis* infection were used for evaluation of sensitivity and specificity of rDgK ELISA using the positive PCR, as a reference, based on the formulae described elsewhere[25]. The analytical specificity of the assays was determined by testing cross-reactivity with serum samples of dogs infected with *Acanthocheilonema reconditum* (n = 25), *Toxocara canis* (n = 4), *Dipylidium caninum* (n = 5), *Leishmania infantum* (n = 5), *Babesia canis* (n = 3), *Ancylostoma caninum* (n =5), and *Dirofilaria repens* (n = 10), all were kindly provided by School of Veterinary Medicine, University of Tehran, Tehran, Iran.

### Statistical analysis

The student’s *t*-test was used to evaluate the significant difference (*P*<0.05) between ODs value of known *D. immitis* positive and negative dog sera in ELISA using GraphPad Prism version 4.0. The concordance between the molecular analysis (standard test) and rDgK ELISA was calculated using the K coefficient (GraphPad software, available online at http://graphpad.com/quickcalcs/kappa1/)

### Bioinformatics analysis

Sequence homology analyses were accomplished using the NCBI databases with BLAST search tool (http://www.ncbi.nlm.nih.gov/). The Geneious software, version 4.8.5, a bioinformatics tool for annotating sequence alignment was used to compare the retrieved sequences.

## RESULTS

### Morphometric and molecular analysis

The mean count of mff for each dog was around 1500 mff/ml of blood samples. The mff of other filariae was not observed. The mean length and the

width of mff were around 300 µm and 6 µm, respectively. The front and rear end of the mff were conical and straight, respectively. The molecular results confirmed the identified *D. immitis* larvae by morphological methods. The BLAST search showed 99% homology of the obtained sequence to *D. immitis* in the GenBank (e.g. AF217800). The amplified sequence was deposited in the GenBank with the accession number KY863453.

### Characterization of DgK gene

The *DgK* transcript was shown to be expressed in adult male and female worms as well as in the mff stage. Sequencing analysis revealed that this gene contained 1092 bp. BLAST analysis of the cloned sequence demonstrated more than 99% sequence identity with the only available sequence of *DgK* cDNA from *D. immitis* in NCBI database (accession number: AB004253). The *DgK* new sequence was deposited in the GenBank with the accession number KU058149. Alignment of the two sequences showed the presence of three nucleotide changes in KU058149, which resulted in two amino acid changes.

### Codon optimization

The sequence of *DgK* gene was optimized based on *E. coli* codon bias. The optimized gene was deposited in the GenBank (accession number: KX434612).

### Characterization of rDgK protein

**Fig. 2:**
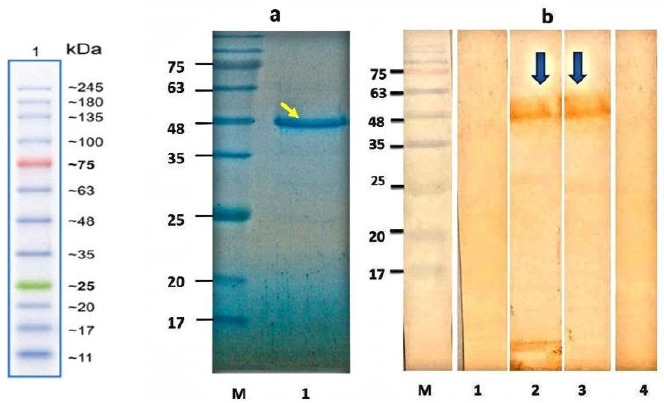
SDS-PAGE analysis of purified antigen and Western blot analysis of rDgK antigen. (a) Purified protein was loaded on 12% acrylamide gel, stained with Coomassie brilliant blue R-250. Lane M, protein molecular standard; lane 1, SDS-PAGE of purified recombinant protein (arrow). (b) Lane M, protein molecular standard; lane 1, Western blotting of expressed rDgK probed with (lane 1) the negative control pool sera, (lane 2) suspected dog pool sera, and (lane 3) the positive-control serum pools (arrows). Lane 4 indicates total protein of untransformed *E. coli* BL21 in Western blot with the positive-control serum pools.

The synthesized gene was subcloned into pET-24a(+) expression vector and the accuracy of cloning procedure was confirmed by restriction enzyme digestion (data not shown). SDS-PAGE analysis of induced bacterial lysate showed the presence of a specific protein band of 50 kDa. rDgK protein was highly purified using a single-step IMAC chromatography (Fig. 2a). After Western blotting, positive pooled sera strongly reacted with purified rDgK, while uninfected pooled sera did not react. There was not any reaction between positive sera and proteins of untransformed BL21 (Fig. 2b).

### Diagnostic potential of rDgK in ELISA

**Fig. 3:**
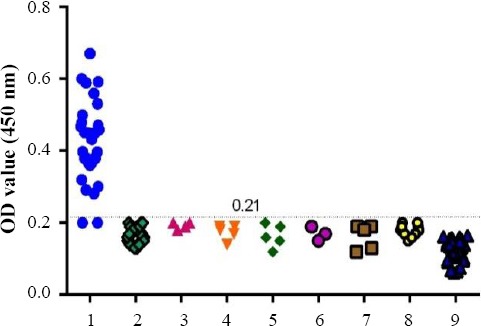
Cross-reactivity of rDgK ELISA. 1, *Dirofilaria immitis*-infected dog sera (n = 30); 2, *A. reconditum* (n = 25); 3, *T. canis* (n = 4); 4, *D. caninum* (n = 5); 5, *L. infantum* (n = 5); 6, *B. canis* (n = 3); 7, *A. caninum* (n = 5); 8, *D. repens* (n = 10); 9, 30 healthy pet dog sera (n = 30).

For evaluation of rDgK antigen, as a serodiagnostic candidate for *D. immitis* infection, a panel of sera comprising positive and negative sera as well as sera from dogs infected with other parasites was used. The cut-off value for negative sera was 0.12. From 30 dog sera infected with *D. immitis*, 28 found to be positive. No false-positive results were observed with sera from healthy pet dogs or other pathogen pool sera, including *A. reconditum*, *T. canis*, *D. caninum, L. infantum*, *B. canis* *A. Caninum*, and *D. repens* related to cross-reactivity test, indicating the specificity of rDgK ELISA for detection of antibody against *D. immitis* (Fig. 3). A significant differences (*P*<0.0001) in the mean OD value between *D. immitis*-infected positive dog sera (0.42 ± 0.11) and healthy dog sera (0.11 ± 0.03) weas found by student’s *t*-test

### Performance of rDgK ELISA

**Fig. 4:**
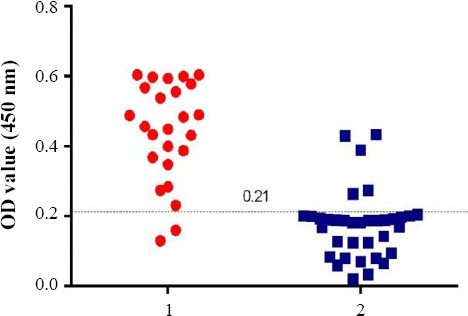
ELISA with rDgK using field dog sera. 1, ELISA value of PCR-positive samples (n = 25); 2, ELISA value of PCR-negative samples (n = 35).

Sixty serum samples were obtained from suspected dogs to verify the performance of the rDgK ELISA in comparison to PCR. Of these serum samples, 25 (41.6%) were found positive by PCR, whereas 28 (46.6%) were positive by rDgK ELISA. On the other hand, 23 (38.3%) samples, which were confirmed to be positive by ELISA, were also positive by PCR. The diagnostic sensitivity of rDgK ELISA was 92.5, when PCR was taken as a reference test. Also, 5 (8.3%) samples, which were negative in PCR, were identified as positive by ELISA (Fig. 4). The diagnostic specificity of rDgK ELISA was 87.5. A significant difference (*P*<0.0001) in the mean OD value between *D. immitis*-infected positive dog sera (0.44 ± 0.02) and uninfected dog sera (0.17 ± 0.01) was found by student’s *t*-test. The results of rDgK ELISA were agreement (0.764) with molecular findings (Table 1).

**Table 1:** Comparison of PCR and rDgK ELISA using suspected field samples

PCR	rDgK ELISA		Total (%)	K coefficient (95%CI)

	Positive (%)	Negative (%)		
Positive	23	2	25 (41.6)	0.764
Negative	5	30	35 (58.3)	
Total	28 (46.6)	32 (53.3)	60 (100)	

### Sensitivity of rDgK ELISA in view of the time points after infection

Based on rDgK ELISA, 2 out of the 15 suspected young dog (<2 years old) were positive, which was consistent with KCT and PCR assays. One of the positive cases was 17 months old, and the other one was 18 months old.

### Finding clinical signs

Most dogs with or without heartworm infection almost had the same moderate clinical sign. In some cases of PCR-positive dogs, the severe clinical signs, including vomiting, cachexia, and coughing, were observed.

## DISCUSSION

In the present study, a new r*DgK* antigen was developed, and its potential value for serodiagnosis of *D. immitis* infection in dogs was evaluated. *D. immitis* infections in dogs are traditionally examined by detection of mff in peripheral blood samples based on KCT[26]. However, some techniques with high sensitivity and specificity are essential; this could be due to low sensitivity of small volume of blood samples testing as well as occult dirofilarisis and operator with high expertise[27]. In recent years, molecular assays such as PCR[26,28], duplex real- time PCR, and multiplex PCR[29] have been developed

ELISA with rDgK using field dog sera. 1, ELISA value of PCR-positive samples (n = 25); 2, ELISA value of PCR-negative samples (n = 35). for identification of *D. immitis* in dogs. However, these methods are expensive and require a skilled technician and specialized laboratory[30].

The results of our study revealed that the sensitivity and specificity of rDgK ELISA were 92.5% and 87.5%, respectively. The diagnostic performance of rDgK is somewhat similar to that represented by *Dg2*[15]. The antigenicity of rDgk was successfully determined by Western blotting. The rDgK ELISA correctly differentiated *D. immitis*-infected positive sera samples from the control-negative sera. In some cases, the samples showed disagreement between PCR and ELISA. Five samples (8.3%) with moderate clinical signs of intolerance to exercise, which showed positivity by ELISA, were negative by PCR. This disparity may be due to occult dirofilariasis characterized by amicrofilaremia[31]. Another observation revealed that 2 positive samples (3.3%; shown by PCR) with coughing signs turned negative by ELISA, which might be due to low heartworm burden[8].

In the current study, it was not possible to evaluate the sensitivity of rDgK ELISA tests in view of worm burden because only samples from owned dogs were included. Therefore, the reason why dogs harboring *D. immitis* microfilariae did not react to rDgK ELISA tests remains doubtful. This type of disagreement between the results of golden standard and ELISA with recombinant antigen had also been reported before[32]. A previous study observed many cases of heartworm infection among younger dogs in endemic areas[33]. We found that the rDgK ELISA was able to diagnose the heartworm infection shortly after infection although more sera are needed to be included for evaluation of rDgK ELISA sensitivity in view of the time points after infection. Due to our limitation of sampling in the present study, the sensitivity of rDgK ELISA in terms of worm counts needs to be evaluated in the next future.

Comprehensive studies have not been performed on the presence of the *Dg2* and *DgK* genes in worms simultaneously. Moreover, more than 90% similarity has been reported between the sequence of their exons and their three-dimensional structure of their respected proteins. So, it seems that the serological tests using rDgK and rDg2 would leads to the same results. However, their efficacy, accuracy, and specificity should be evaluated in the future studies on clinical samples. To the best of our knowledge, this study is the first report on the use of r*DgK* in ELISA format for the diagnosis of *D. immitis* infection in dogs. Due to its antigenic potential, this recombinant protein can be used for diagnosis of canine dirofilariasis.
